# Back Numbers Wanted

**Published:** 1884-03

**Authors:** 


### BACK NUMBERS WANTED.

The editions of the INDEPENDENT PRACTITIONER for August, 1883, and January, 1884, have been entirely exhausted. There must be many numbers in the hands of dentists who do not care to preserve them. To any one who will return to us either of the numbers mentioned, we will mail a copy of CAULK'S DENTAL ANNUAL, a very valuable publication, containing statistics and information that one must search through whole libraries to obtain.

